# Biomarker-guided acute kidney injury risk assessment under liberal versus restrictive fluid therapy - the prospective-randomized MAYDAY-trial

**DOI:** 10.1038/s41598-024-68079-2

**Published:** 2024-07-24

**Authors:** Alexandra M. Anker, Marc Ruewe, Lukas Prantl, Magnus Baringer, Michael T. Pawlik, Florian Zeman, Ivan Goecze, Silvan M. Klein

**Affiliations:** 1 Department of Plastic, Reconstructive, Aesthetic, and Hand Surgery, University Hospital Regensburg, University of Regensburg, Franz-Josef-Strauß-Allee 11, 93053 Regensburg, Germany; 2 Department of Anaesthesiology and Intensive Care Medicine, Caritas Hospital St. Josef, Landshuter Str. 65, 93053 Regensburg, Germany; 3 Center for Clinical Studies, University Hospital Regensburg, University of Regensburg, Franz-Josef-Strauß-Allee 11, 93053 Regensburg, Germany; 4 Department of Surgery and Operative Intensive Care, University Hospital Regensburg, University of Regensburg, Franz-Josef-Strauß-Allee 11, 93053 Regensburg, Germany

**Keywords:** Acute kidney injury, Intraoperative fluid management, Fluid therapy, Breast neoplasms/surgery, Breast reconstruction, Urinary biomarkers, Biomarkers, Randomized controlled trials, Kidney, Kidney diseases

## Abstract

Acute kidney injury (AKI) prevalence in surgical patients is high, emphasizing the need for preventative measures. This study addresses the insufficient evidence on nephroprotective intraoperative fluid resuscitation and highlights the drawbacks of relying solely on serum creatinine/urine output to monitor kidney function. This study assessed the impact of intraoperative fluid management on AKI in female breast cancer patients undergoing autologous breast reconstruction, utilizing novel urinary biomarkers (TIMP-2 and IGFBP-7). In a monocentric prospective randomized controlled trial involving 40 patients, liberal (LFA) and restrictive (FRV) fluid management strategies were compared. TIMP-2 and IGFBP-7 biomarker levels were assessed using the NephroCheck (bioMerieux, France) test kit at preoperative, immediate postoperative, and 24-h postoperative stages. FRV showed significantly higher immediate postoperative biomarker levels, indicating renal tubular stress. FRV patients had 21% (4/19) experiencing AKI compared to 13% (2/15) in the LFA group according to KDIGO criteria (p = 0.385). Restrictive fluid resuscitation increases the risk of AKI in surgical patients significantly, emphasizing the necessity for individualized hemodynamic management. The findings underscore the importance of urinary biomarkers in early AKI detection.

## Introduction

The prevalence of acute kidney injury (AKI) in hospitalized patients is alarmingly high, ranging from 2 to 18%^1,2^. Recent literature has raised awareness about the underestimated danger of postoperative AKI in surgical patients^3–6^. Overly restricted or inadequate intraoperative fluid resuscitation may lead to insufficient intravascular volume and reduced renal perfusion pressure, which is one major cause of AKI^3,7–10^. In contrast, restrictive fluid regimes have been proposed as beneficial in terms of surgical outcome in microsurgery^11,12^. However, there is still a lack of supporting evidence to provide clear guidance on nephroprotective intraoperative hemodynamic management.

Elevations in serum creatinine might only become noticeable after substantial kidney damage, leading to a delayed diagnosis of postoperative AKI^2,13,14^. To address the drawbacks associated with serum creatinine as a metric and to encourage early detection of AKI, recent studies have focused on identifying novel biomarkers. Urine markers such as inhibitor of metalloproteinase-2 (TIMP-2) and insulin-like growth factor binding protein 7 (IGFBP-7) predict renal tubular stress with high sensitivity and have been proven to be a reliable indicator for imminent AKI at a preclinical stage^3,15–19^.

This is the first clinical study that monitors the impact of intraoperative fluid resuscitation on renal function based on the newly implemented biomarkers TIMP-2 and IGFBP-7. The effects of two standardized hemodynamic strategies involving either liberal or restricted fluid administration were examined in a consistent group of female breast cancer patients undergoing free flap breast reconstruction within a prospective randomized setup.

## Methods

### Trial design

A randomized assessor-blinded trial was designed to compare a liberal (LFA) to a restrictive (FRV) intraoperative fluid management approach.

The study protocol was approved by the ethics committee of the University of Regensburg (reference 16-293-101, amendment 18/08/2018) and was registered on the German Clinical Trial Register database (reference DRKS00017735, registered 07/08/2019). The study was conducted in accordance with the ethical standards of the Helsinki Declaration of 1975.

### Patient selection and randomization

Female breast cancer patients undergoing autologous breast reconstruction with the deep inferior epigastric perforator (DIEP) free flap following mastectomy were considered eligible for voluntary participation in the study at the study center. Informed consent was obtained from all participants. Both primary (simultaneous to mastectomy) and delayed DIEP flap reconstructions were included. The flaps were harvested according to the trial center’s standard by equally qualified consultant plastic surgeons^20–22^.

Patients with a self-reported or documented AKI, chronic kidney disease, kidney transplantation or heart failure were excluded based on comprehensive history and thorough review of all available laboratory results, medical records, discharge summaries, and diagnosis lists from previous admissions. Patients with serum creatinine levels > 1.0 mg/dl in the 48-h preoperative time window, were excluded from the study.

All participants were recruited from August 2019 to July 2021.

### Trial treatments

The standardized hemodynamic regimes were established and reported previously^20,22^.

In the LFA group, a bolus of balanced crystalloid solution was administered at a dose of 10 ml per kilogram (kg) of bodyweight during anesthesia induction and a maximum of 8 ml per kilogram per hour during the surgery.

The FRV regime aimed to achieve a net zero fluid balance, with no fluid bolus administered during anesthesia induction. Balanced crystalloid solution was administered at a maximum rate of 5 ml per kilogram per hour intraoperatively.

Crystalloid solutions administered were Sterofundin 1/1 E ISO in both groups. Colloid solutions or stored blood could be administered in both groups to replace blood loss in a 1:1 ratio. Colloid solutions used for blood loss replacement in both groups were Gelafundin ISO 40 mg/ml. The transfusion threshold for hemoglobin value was set at < 6 g/dl. Urinary excretion was balanced at a ratio of 1:1 independently of the hemodynamic regime. In both groups the mean arterial pressure (MAP) was consistently maintained between 65 and 85 mmHg throughout the surgery, assessed via an arterial line.

All other intra- and perioperative care was performed equally in both groups according to the trial center’s standard.

Anesthesia induction was routinely performed with propofol (2 mg/kg bodyweight) and sufentanil (0.3 µg/kg bodyweight). Orotracheal intubation was facilitated with atracurium (0.5 mg/kg bodyweight). Anesthesia was maintained using sevoflurane (minimum alveolar concentration = 0.8) and intermittent bolus administration of sufentanil (10 µg). Crystalloid solutions and norepinephrine were administered by continuous infusion throughout the surgery via central or peripheral venous access.

### Trial outcomes and patient follow-up

The primary study outcome were [TIMP-2]⋅[IGFBP-7] urinary biomarker levels. [TIMP-2]⋅[IGFBP-7] baseline levels were assessed preoperatively on the day of surgery immediately before anesthesia induction (t0). Further samples were taken from foley catheter urine immediately postoperatively (t1) as well as 24 h later in the morning of the first postoperative day (t2). The commercially available Nephrocheck test kit (bioMerieux, France) was used for [TIMP-2]⋅[IGFBP-7] assessment. As immediate sample analysis was not feasible at the study center, samples were frozen at − 20 °C after centrifugation and stored. Frozen samples were thawed and warmed to room temperature and the Nephrocheck test (bioMérieux, France) was performed immediately using the ASTUTE 140 Meter^23^.

Secondary outcomes included acute kidney injury, postoperative serum creatinine and GFR (measured within a 48-h postoperative period), intraoperative urine output, intraoperative oliguria (defined as < 0.5 ml/h/kg body weight), renal replacement therapy within a follow-up of six weeks postoperatively, and duration of hospitalization. Acute kidney injury was assessed in a 48-h postoperative time window and defined according to the standardized “Kidney Disease: Improving Global Outcome” (KDIGO) criteria which consider both serum creatinine changes and oliguria^24^.

### Statistical analysis

A priori sample size calculation was performed for a two-sided unpaired t-Test with regard to the primary outcome measure assuming a power of 0.8, an effect size of 0.6, and a level of significance of 0.05. Forty-five patients per group were calculated. After including 20 patients per group, an interim analysis was conducted based on the recommendations for data and safety monitoring^25^.

Metric data are expressed as mean ± standard deviation (SD) for normally distributed variables, and as median (interquartile range, IQR) for non-normally distributed variables. Statistical significance for normally distributed data was analyzed using parametric unpaired Student’s t-tests. For non-normally distributed data, non-parametric two-sided Fisher’s exact tests and Mann–Whitney U tests were performed.

A value of p < 0.05 was considered statistically significant. Statistical analysis was performed using SPSS version 26.0 (IBM Corporation, Armonk, NY, USA). The Sankey plot was performed with R software (Integrated Development for R, 2021, PBC, Boston, MA, USA).

## Results

### Patient enrollment and follow-up

**Figure 1:**
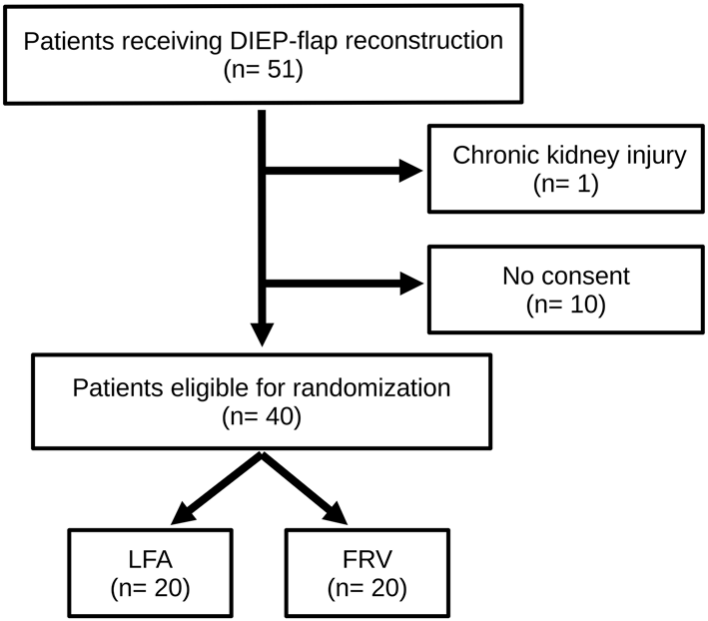
Study flow diagram. During the inclusion period, 51 patients who were scheduled for breast reconstruction with DIEP flaps were screened at the study center. Of these, 40 patients were randomized to the two study groups LFA and FRV. *LFA* liberal fluid administration, *FRV* fluid restriction vasopressor support.

Forty patients meeting the eligibility criteria were enrolled. Of these, 20 participants were randomly assigned to the LFA and 20 to the FRV regime, respectively (Fig. 1).

For ethical reasons recruitment was terminated upon reaching 20 participants per study arm, as the interim analysis revealed highly significant differences between the treatment groups at this stage of enrollment. Six week follow-up was completed by all study participants (n = 40). Patient demographic characteristics, duration of surgery and hospitalization were homogeneously distributed across the study populations (Table 1).

**Table 1:** Patient demographics and perioperative characteristics.

	LFA	FRV	*p*-value
Age (years) (median, IQR)	51 (16)	50 (13)	0.478
BMI (kg/m^2^) (mean ± SD)	26.4 ± 5.2	26.3 ± 3.1	0.959
ASA II score status (n)	20 (100%)	20 (100%)	
Hospitalization (days) (median, IQR)	6 (3)	6 (2)	0.640
Duration of surgery (min) (median, IQR)	315 (197)	319 (180)	0.414
Immediate reconstruction (n)	4 (20%)	8 (40%)	0.301
Delayed reconstruction (n)	16 (80%)	12 (60%)	0.301

*LFA* liberal fluid administration, *FRV* fluid restriction vasopressor support, *ASA* American Society of Anesthesiologists, *BMI* body mass index.

### Trial treatment

**Figure 2:**
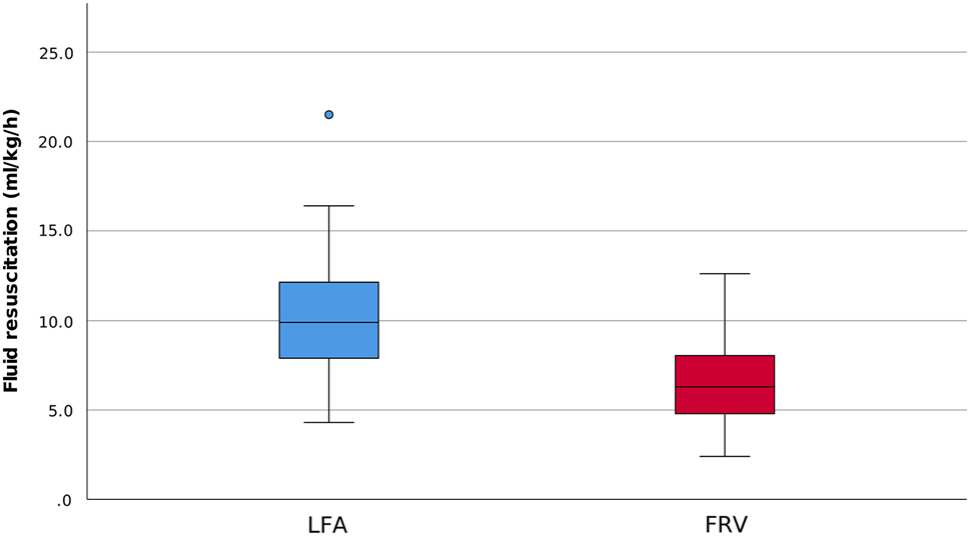
Fluid resuscitation strategies. Median fluid infusion (IQR) rates showed highly statistically significant differences between the LFA (9.9 (7.5) ml/kg/h) and FRV (6.3 (3.6) ml/kg/h) groups (p < 0.001). *LFA* liberal fluid administration, *FRV* fluid restriction vasopressor support.

Mean fluid resuscitation rates during surgery in each group are presented in Fig. 2. Intraoperatively, no blood transfusions were administered.

### Primary outcome

**Figure 3:**
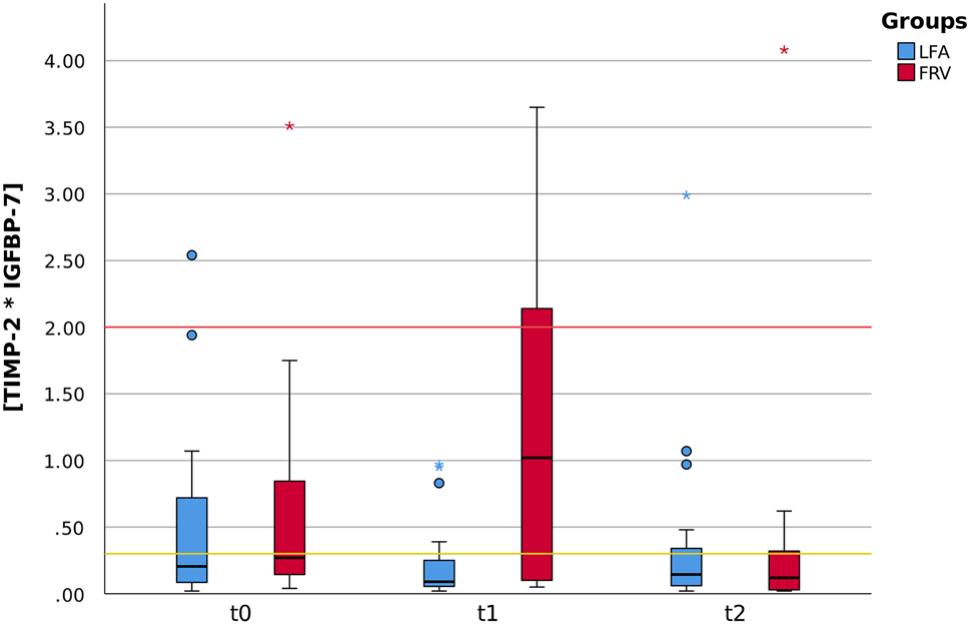
Box plots display [TIMP-2]⋅[IGFBP-7] median biomarker levels at t0 (baseline level before anesthesia induction), t1 (immediately postoperatively) and t2 (first postoperative day). Immediately postoperative median t1 levels were statistically significantly higher in the FRV cohort (t1_LVA_ 0.09 (0.24), (n = 19); t1_FRV_ 1.02 (2.18), (n = 18); p = 0.002). The yellow line at 0.3 and the red line at 2.0 correspond to the cutoff thresholds indicating low and high risk for AKI, respectively. Risk thresholds were defined according to the current evidence-based recommendations of the manufacturer Nephrocheck (bioMerieux, France). *LFA* liberal fluid administration, *FRV* fluid restriction vasopressor support.

Urinary biomarker levels assessed at t0, t1 and t2 under the impact of the defined intraoperative fluid management strategies LFA and FRV are shown in Table 2 and Fig. 3. While median baseline (t0) as well as median 24 h postoperative [TIMP-2]⋅[IGFBP-7] levels (t2) were similar throughout both study groups, immediately postoperative median t1 levels were statistically significantly higher in the FRV cohort indicating immediate renal tubular stress (Table 2).

**Table 2:** Primary outcome. [TIMP-2]⋅[IGFBP-7] median (IQR) biomarker levels at t0 (baseline level before anesthesia induction), t1 (immediately postoperatively) and t2 (first postoperative day).

	LFA	FRV	p-value
	[TIMP-2]⋅[IGFBP-7]	[TIMP-2]⋅[IGFBP-7]	
t0	0.21 (0.74) (n = 20)	0.27 (0.7) (n = 20)	0.301
t1	0.09 (0.24) (n = 19)	1.02 (2.18) (n = 18)	**0.002**
t2	0.15 (0.3) (n = 20)	0.12 (0.3) (n = 18)	0.478

*LFA* liberal fluid administration, *FRV* fluid restriction vasopressor support.

Significant values are in bold.

**Figure 4:**
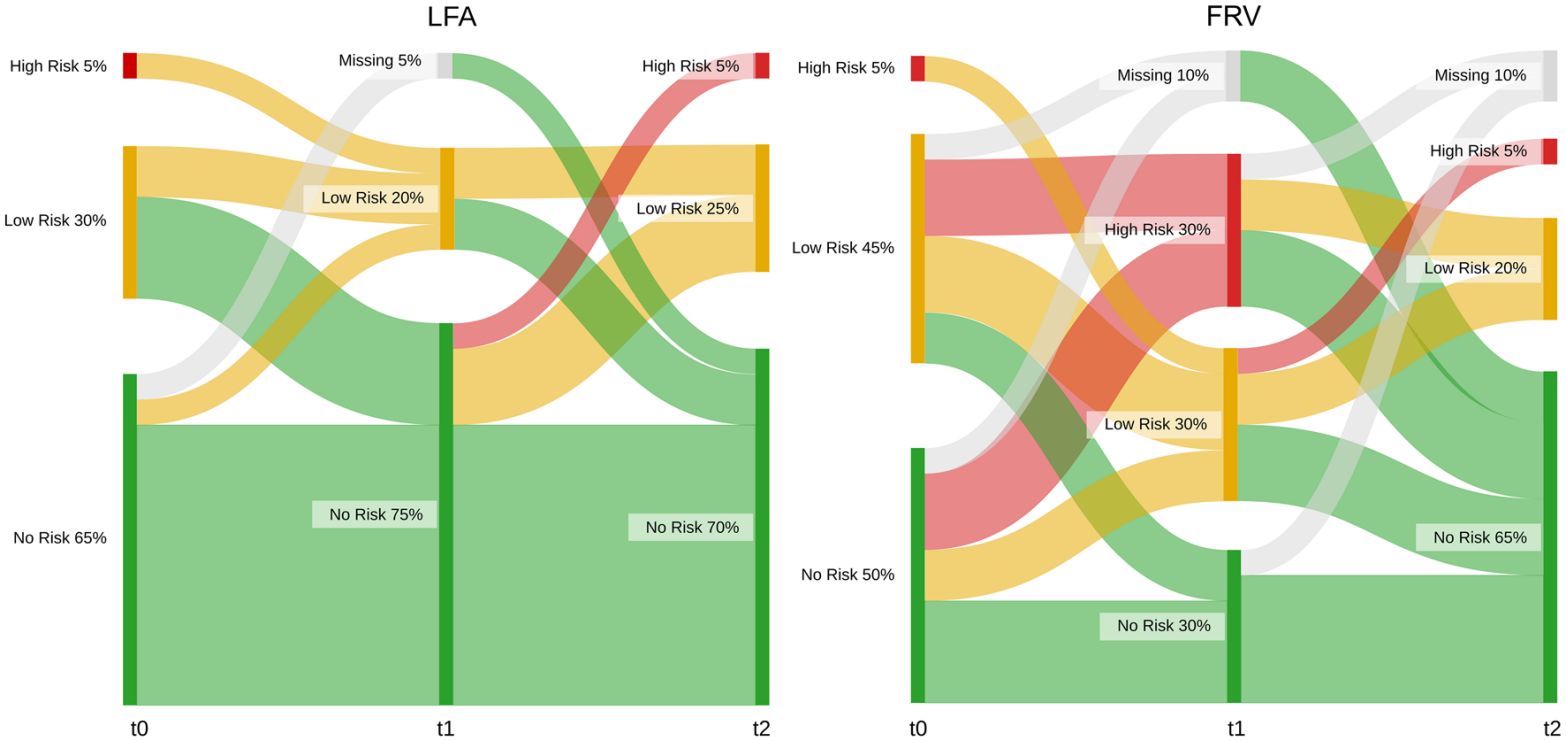
The Sankey diagram displays [TIMP-2]⋅[IGFBP-7] biomarker dependent AKI risk stratification of study participants in each group over time (t0–t2). t0 corresponds to the baseline biomarker level before anesthesia induction, t1 is immediately postoperatively and t2 is on the first postoperative day. The different colors indicate the risk for AKI with *no risk* shown in green, *low risk* in yellow, and *high risk* in red. The color grey represents missing data. *LFA* liberal fluid administration, *FRV* fluid restriction vasopressor support.

The Sankey plot in Fig. 4 visualizes [TIMP-2]⋅[IGFBP-7] dependent AKI risk stratification of study participants in each group over time (t0–t2).

The cut-off values indicating low-risk (0.3) and high-risk (2.0) for the development of a clinically manifest AKI were chosen according to the current recommendations of the manufacturer Nephrocheck (bioMerieux, France) providing the AKI risk assessment tool based on the biomarkers [TIMP-2]⋅[IGFBP-7]. While 5% (1/20) of patients in each group had high AKI risk at the baseline measurement, fluid restriction was associated with 30% (6/20) of participants experiencing high-risk for AKI at t1. Of note, none of the patients receiving liberal fluid resuscitation exceeded the 2.0 cut-off value. Interestingly, biomarker levels returned to normal during the 24-h postoperative period (t2) with—corresponding to the preoperative setting (t0)—again 5% (1/20) of patients showing high AKI risk independent of the study group. No specific nephroprotective measures were applied in the immediate postoperative period in this study’s cohort. Patients were treated according to the trial center’s postoperative standard protocol regardless of the applied intraoperative fluid strategy.

### Secondary outcomes

Table 3 summarizes the secondary outcomes of the study. AKI occurred in 21% (4/19) in the FRV group and 13% (2/15) in the LFA group, according to the KDIGO criteria that consider serum creatinine changes and oliguria. None of the patients required renal replacement therapy within 6 weeks postoperatively, regardless of the intraoperative fluid strategy.

**Table 3:** Secondary outcomes.

	LFA	FRV	p-value
Crea pre-OP* (mg/dl) (median, IQR)	0.73 (0.19) (n = 19)	0.68 (0.09) (n = 19)	0.223
Crea post-OP* (mg/dl) (median, IQR)	0.69 (0.16) (n = 14)	0.65 (0.16) (n = 13)	0.220
eGFR pre-OP* (ml/min) (median, IQR)	93 (27) (n = 19)	104 (13) (n = 19)	**0.032**
eGFR post-OP* (ml/min) (median, IQR)	100 (23) (n = 14)	104 (15) (n = 14)	0.350
Hb pre-OP (g/dl) (mean ± SD)	13.1 ± 1 (n = 19)	12.9 ± 1.1	0.662
Hb post-OP (g/dl) (mean ± SD)	10 ± 1.1 (n = 19)	9.9 ± 1.4	0.781
Urine output intra-OP (ml/kg/h) (median, IQR)	2.2 (3.4) (n = 15)	0.7 (1.16) (n = 19)	**0.002**
Oliguria (< 0.5 ml/kg/h) intra-OP (n)	1 (7%, n = 15)	4 (21%, n = 19)	0.355
AKI** (n)	2 (13%, n = 15)	4 (21%, n = 19)	0.385
Norepinephrine administration rate (µg/kg/min) (median, IQR)	0.038 (0.097) (n = 16)	0.077 (0.070) (n = 19)	0.088

*LFA* liberal fluid administration, *FRV* fluid restriction vasopressor support.

*Postoperative serum creatinine and eGFR levels measured within a 48 h time window. Preoperative serum creatinine and eGFR levels were measured within a 48 h preoperative time window.

**Acute kidney injury (AKI) was defined according to the standardized “Kidney Disease: Improving Global Outcome” (KDIGO) criteria^24^. Due to technical and logistical difficulties, some data were not collected*.* These missing data are discussed in the “Discussion” section of the manuscript. Please see the “Discussion” section of the manuscript for further details.

Significant values are in bold.

## Discussion

This study investigated the risk of kidney injury using novel urinary renal stress markers in 40 female patients undergoing autologous breast reconstruction with the DIEP flap, under the influence of restrictive vs. liberal intraoperative fluid administration strategies.

The DIEP flap procedure is the worldwide most frequently applied method of autologous oncologic breast reconstruction and is therefore a predestined study model due to a number of reasons. Firstly, patients undergoing DIEP flap breast reconstructions are typically female, middle-aged, moderately obese, and in good general health, which provides a homogeneous cohort. Secondly, the DIEP flap procedure is highly consistent in its operative steps, resulting in comparable operation times, which allows for uniform study conditions. Furthermore, the hemodynamic regimes investigated in this study had already been established in a previous cohort of DIEP flap patients, which ensured a well-established protocol for the current study^20,22^. In addition, cancer patients, due to the prolonged duration of the disease and oncologic therapies, are at an increased risk of developing AKI. This risk is particularly high for breast cancer patients with triple-negative tumor types, as current treatment guidelines generally require the use of Cisplatin as a first-line chemotherapeutic agent^26^. Cisplatin is a known nephrotoxic agent, which is associated with the development of AKI^27^. Therefore, the avoidance of intraoperative hit phenomena to the kidney has highest priority in this specifically vulnerable patient population to prevent permanent injury^28^.

However, fluid restriction has been found to enhance DIEP flap microperfusion, while LFA stands as a significant independent predictor of complications in free flap reconstruction^12,20^. In fact, the latest evidence-based recommendations favored a fluid-restrictive regimen over the indiscriminate administration of fluid in DIEP flap breast reconstruction^11,12,20^**.**

In this trial 15% of patients were diagnosed with AKI according to the KDIGO criteria, with twice as many events in the FRV (n = 4) than in the LFA (n = 2) group. While a recent prospective, observational, multi-center study in 30 countries reported an 18.4% incidence of AKI in patients over all surgical specialties undergoing major surgery (> 2 h), there is limited evidence regarding AKI incidence rates in complex soft tissue reconstructions such as the DIEP flap^5,6^. DIEP flap breast reconstructions involve prolonged operation times (averaging 5–6 h) and large thoracic and abdominal soft tissue wound cavities with consecutive fluid shift into third space^20,22^. With regard to these circumstances, the incidence rates of AKI observed in our current study are likely to be comparable to those reported for major surgery from other fields^4,5^. Once more, these findings underline the need for nephroprotective measures, such as optimized intraoperative fluid resuscitation, for any extended surgical procedure.

The observed sensitive urinary biomarker levels indicating stress of the renal tubular epithelium well before the elevation of creatinine serum levels^29,30^. Although serum creatinine still is the most widely used parameter in clinical practice to assess renal function, its use has been debated due to limitations in its sensitivity and specificity for several years^13,15,31^. Creatinine levels may not increase until there is significant damage to the kidney, resulting in delayed detection of renal dysfunction^13,32^. To overcome the limitations of serum creatinine as a marker of renal function, novel biomarkers, such as the highly-sensitive [TIMP-2]⋅[IGFBP-7], have been proposed to improve the early detection and diagnosis of AKI^19,29,33,34^. In cardiac and major abdominal surgery patients with high-risk for AKI development based on postoperative [TIMP-2]⋅[IGFBP-7] assessment, the consecutive implementation of nephroprotective measures (“KDIGO care bundle”) in randomized-controlled trials succeeded to significantly reduce postoperative AKI II/III events and severity^9,35^. Accentuated by a recently published metaanalysis, these studies highlight the capability of the newly implemented biomarkers to serve as early indicators of AKI, ultimately resulting in improvements in patient outcomes^18^.

In the current study [TIMP-2]⋅[IGFBP-7] levels were assessed under the impact of two defined intraoperative fluid management strategies LFA and FRV. While baseline (t0) as well as 24 h postoperative (t2) biomarker levels were comparable throughout both study groups, immediate postoperative (t1) levels were statistically significantly higher in the FRV cohort in direct comparison to the LFA regime. Hence, fluid restriction was associated with kidney stress. The findings of this biomarker-based approach support the hypothesis of intraoperative hypovolemia as a major risk factor for AKI in surgical patients^3,7–9^.

One possible explanation for the spontaneous postoperative regression of [TIMP-2]⋅[IGFBP-7] levels might be an activation of the renal functional reserve capacity in response to tubular stress. It has been suggested that the kidney organ is capable of recruiting functional reserve capacities in response to physiological demands, serving as a preventive measure against imminent AKI. Before the basal glomerular filtration rate (GFR) starts to decrease in subclinical kidney damage, it is believed that the kidney relies on its functional reserve to maintain proper function. Therefore, the renal system provides extra capacity to compensate for any early signs of damage^30,36^. Accordingly, among patients undergoing cardiac surgery, elevated postoperative biomarkers have recently been linked to a reduction in renal function recovery at three months follow-up, even though serum creatinine levels had returned to normal. In contrast, patients with normal biomarker levels after surgery did not show any decline in renal function recovery^30^.

As expected prior to surgery [TIMP-2]⋅[IGFBP-7] baseline levels (t0) did not show statistically significant differences among the study groups LFA and FRV. A conspicuous number of patients, however, exhibited elevated biomarker levels (> 0.3), signifying an increased risk of AKI at the initiation of the surgical procedure. This coincides with the standard six-hour preoperative fasting period that patients routinely undergo at the study center to mitigate the risk of gastric content aspiration during anesthesia induction. It is supposed that prolonged preoperative fasting induces hypovolemia^37^. Likely, sensitive renal biomarkers might mirror this dehydrated hemodynamic status in certain patients.

Overall, on immediate postoperative [TIMP-2]⋅[IGFBP-7] assessment (t1), the risk of AKI was considered high in 30% and low in 30% of cases in the FRV group. In contrast, none of the patients in the LFA group had a high risk, and only 20% yielded a low risk for AKI.

Notably, according to the currently established KDIGO criteria, which consider GFR as measured by serum creatinine and quantitative diuresis, a considerably lower number of patients (13% (2/15) of AKI under LFA, 21% (4/19) under FRV support) would have been effectively diagnosed as AKI. On the one hand, this discrepancy rekindles the ongoing debate regarding the use of serum creatinine/GFR as the standard parameters to assess kidney function as discussed above. On the other hand, it remains unclear to date, whether an isolated elevation in biomarkers, in the absence of changes in serum creatinine/oliguria, truly correlates with long-term renal or general complications^15^.

This study has some notable limitations. Due to its monocentric design, the findings may lack generalizability and the data may not be universally applicable to any other population. Another limitation is that the trial specified the administration of fluid therapy during surgery, but there was no control over postoperative fluid turnover, which may have impacted t2 biomarker levels. The reason for this limitation is within the nature of large wound surfaces, that cause fluid loss by wound secretion into wound dressings, that are ultimately difficult to assess. However, it is important to note that a main objective of the t2 measurement was to gain insight into the general dynamics of [TIMP-2]⋅[IGFBP-7].

As the study focused on the perioperative setting, potential middle or long-term effects of the hemodynamic regimes applied here or the consequences of a singular [TIMP-2]⋅[IGFBP-7] elevation are beyond the scope. Future studies should investigate whether an increase in biomarkers is associated with any long-term renal or general complications.

A limitation of this study is the presence of incomplete datasets for the secondary outcomes, primarily due to technical and logistical challenges encountered during data collection. For example, there were instances where the Foley catheter bag was emptied by the anesthesiologist on duty without proper documentation, leading to missing urine output data. Additionally, the study was not extended further to collect more valid data for secondary outcomes, given the conclusive nature of the primary outcomes. These factors contribute to the limitations of the study and highlight the need for more rigorous data collection protocols in future research.

Another limitation is the slight, yet statistically significant difference in preoperative eGFR values between the LFA and FRV groups, despite randomization. To address this issue, a correlation analysis was conducted between the preoperative eGFR values and immediately postoperative t1 biomarker levels, which revealed no significant correlation (see also Supplementary Material 1).

This is the first randomized controlled study monitoring intraoperative [TIMP-2]⋅[IGFBP-7] biomarker levels under the impact of two different fluid management strategies. The study findings indicate that restrictive intraoperative fluid resuscitation is associated with acute and temporary kidney tubular stress. These findings should be further investigated in larger multi-center trials.

## Conclusion

Our study found higher renal stress biomarkers post-surgery in the FRV group compared to LFA, suggesting restrictive fluid resuscitation may increase AKI risk and highlighting the need for individualized hemodynamic management. While further studies with larger sample size are needed to elucidate the clinical relevance of a singular and acute [TIMP-2]⋅[IGFBP-7] elevation, current fluid restriction recommendations for DIEP flap procedures should be critically reconsidered.

### Supplementary Information

Supplementary Information.

## Data Availability

Data supporting the results reported in the article can be requested from the corresponding author.

## References

[1] Lewington, A. J., Cerda, J. & Mehta, R. L. Raising awareness of acute kidney injury: A global perspective of a silent killer. *Kidney Int.* **84**, 457–467 (2013). 23636171 10.1038/ki.2013.153 PMC3758780

[2] Bellomo, R., Kellum, J. A. & Ronco, C. Acute kidney injury. *Lancet* **380**, 756–766 (2012). 22617274 10.1016/S0140-6736(11)61454-2

[3] Raphael Weiss, M. M., Pavenstädt, H.-J. & Zarbock, A. Acute kidney injury—A frequently underestimated problem in perioperative medicine. *Dtsch. Arztebl. Int.* **1**, 833–842 (2019). 10.3238/arztebl.2019.0833 PMC6962766 31888797

[4] O’Connor, M. E., Kirwan, C. J., Pearse, R. M. & Prowle, J. R. Incidence and associations of acute kidney injury after major abdominal surgery. *Intens. Care Med.* **42**, 521–530 (2016). 10.1007/s00134-015-4157-7 26602784

[5] Zarbock, A. *et al.* Epidemiology of surgery associated acute kidney injury (EPIS-AKI): A prospective international observational multi-center clinical study. *Intens. Care Med.*10.1007/s00134-023-07169-7 (2023). 10.1007/s00134-023-07169-7 PMC10709241 37505258

[6] Sung, H. M. *et al.* Association between postoperative acute kidney injury and mortality after plastic and reconstructive surgery. *Sci. Rep.* **12**, 20050 (2022). 36414767 10.1038/s41598-022-24564-0 PMC9681753

[7] Myles, P. S. *et al.* Restrictive versus liberal fluid therapy for major abdominal surgery. *N. Engl. J. Med.* **378**, 2263–2274 (2018). 29742967 10.1056/NEJMoa1801601

[8] Schmid, S. *et al.* Algorithm-guided goal-directed haemodynamic therapy does not improve renal function after major abdominal surgery compared to good standard clinical care: A prospective randomised trial. *Crit Care* **20**, 50 (2016). 26951105 10.1186/s13054-016-1237-1 PMC4782303

[9] Gocze, I. *et al.* Biomarker-guided intervention to prevent acute kidney injury after major surgery: The prospective randomized BigpAK study. *Ann. Surg.* **267**, 1013–1020 (2018). 28857811 10.1097/SLA.0000000000002485

[10] Sun, L. Y., Wijeysundera, D. N., Tait, G. A. & Beattie, W. S. Association of intraoperative hypotension with acute kidney injury after elective noncardiac surgery. *Anesthesiology* **123**, 515–523 (2015). 26181335 10.1097/ALN.0000000000000765

[11] Karamanos, E., Walker, R., Wang, H. T. & Shah, A. R. Perioperative fluid resuscitation in free flap breast reconstruction: When is enough enough? *Plast. Reconstr. Surg. Glob. Open* **8**, e2662 (2020). 32537330 10.1097/GOX.0000000000002662 PMC7253255

[12] Motakef, S., Mountziaris, P. M., Ismail, I. K., Agag, R. L. & Patel, A. Emerging paradigms in perioperative management for microsurgical free tissue transfer: Review of the literature and evidence-based guidelines. *Plast. Reconstr. Surg.* **135**, 290–299 (2015). 25539313 10.1097/PRS.0000000000000839

[13] Perrone, R. D., Madias, N. E. & Levey, A. S. Serum creatinine as an index of renal function: New insights into old concepts. *Clin. Chem.* **38**, 1933–1953 (1992). 1394976 10.1093/clinchem/38.10.1933

[14] Mårtensson, J., Martling, C.-R. & Bell, M. Novel biomarkers of acute kidney injury and failure: Clinical applicability. *Br. J. Anaesth.* **109**, 843–850 (2012). 23048068 10.1093/bja/aes357

[15] Ostermann, M. *et al.* Recommendations on acute kidney injury biomarkers from the acute disease quality initiative consensus conference: A consensus statement. *JAMA Netw. Open* **3**, e2019209 (2020). 33021646 10.1001/jamanetworkopen.2020.19209

[16] Joannidis, M. *et al.* Use of cell cycle arrest biomarkers in conjunction with classical markers of acute kidney injury. *Crit. Care Med.* **47**, e820–e826 (2019). 31343478 10.1097/CCM.0000000000003907 PMC6750148

[17] Ostermann, M. *et al.* Kinetics of urinary cell cycle arrest markers for acute kidney injury following exposure to potential renal insults. *Crit. Care Med.* **46**, 375–383 (2018). 29189343 10.1097/CCM.0000000000002847 PMC5821475

[18] Li, Z., Tie, H., Shi, R., Rossaint, J. & Zarbock, A. Urinary [TIMP-2]·[IGFBP7]-guided implementation of the KDIGO bundle to prevent acute kidney injury: A meta-analysis. *Br. J. Anaesth.* **128**, e24–e26 (2022). 34794767 10.1016/j.bja.2021.10.015

[19] Obata, Y., Kamijo-Ikemori, A., Shimmi, S. & Inoue, S. Clinical usefulness of urinary biomarkers for early prediction of acute kidney injury in patients undergoing transaortic valve implantation. *Sci. Rep.* **13**, 18569 (2023). 37903844 10.1038/s41598-023-46015-0 PMC10616062

[20] Anker, A. M. *et al.* Assessment of DIEP flap perfusion with intraoperative indocyanine green fluorescence imaging in vasopressor-dominated hemodynamic support versus liberal fluid administration: A randomized controlled trial with breast cancer patients. *Ann. Surg. Oncol.* **27**, 399–406 (2020). 31468214 10.1245/s10434-019-07758-1

[21] Anker, A. M. *et al.* Clinical impact of DIEP flap perforator characteristics—A prospective indocyanine green fluorescence imaging study. *J. Plast. Reconstr. Aesthetic Surg.* **73**, 1526–1533 (2020). 10.1016/j.bjps.2020.01.019 32507580

[22] Anker, A. M. *et al.* Vasopressor support vs liberal fluid administration in deep inferior epigastric perforator (DIEP) free flap breast reconstruction—A randomized controlled trial. *Clin. Hemorheol. Microcirc.* **69**, 37–44 (2018). 29660924 10.3233/CH-189129

[23] Pajenda, S. *et al.* NephroCheck data compared to serum creatinine in various clinical settings. *BMC Nephrol.* **16**, 206 (2015). 26651477 10.1186/s12882-015-0203-5 PMC4674950

[24] Khwaja, A. KDIGO clinical practice guidelines for acute kidney injury. *Nephron Clin. Pract.* **120**, c179–c184 (2012). 22890468 10.1159/000339789

[25] Ciolino, J. D., Kaizer, A. M. & Bonner, L. B. Guidance on interim analysis methods in clinical trials. *J. Clin. Transl. Sci.* **7**, e124 (2023). 37313374 10.1017/cts.2023.552 PMC10260346

[26] Lu, F. *et al.* Efficacy and safety of platinum-based chemotherapy as first-line therapy for metastatic triple-negative breast cancer: A meta-analysis of randomized controlled trials. *Technol. Cancer Res. Treat.* **20**, 15330338211016368 (2021). 33977814 10.1177/15330338211016369 PMC8120541

[27] McSweeney, K. R. *et al.* Mechanisms of cisplatin-induced acute kidney injury: Pathological mechanisms, pharmacological interventions, and genetic mitigations. *Cancers* **13**, 1572 (2021). 33805488 10.3390/cancers13071572 PMC8036620

[28] Anker, A. M., Prantl, L. & Klein, S. M. ASO author reflections: The silent force behind microsurgery. *Ann. Surg. Oncol.* **27**, 407–408 (2020). 31667724 10.1245/s10434-019-08001-7

[29] Kashani, K. *et al.* Discovery and validation of cell cycle arrest biomarkers in human acute kidney injury. *Crit. Care Lond. Engl.* **17**, R25 (2013). 10.1186/cc12503 PMC4057242 23388612

[30] Husain-Syed, F. *et al.* Persistent decrease of renal functional reserve in patients after cardiac surgery-associated acute kidney injury despite clinical recovery. *Nephrol. Dial. Transplant.* **34**, 308–317 (2019). 30053231 10.1093/ndt/gfy227

[31] Siew, E. D., Ware, L. B. & Ikizler, T. A. Biological markers of acute kidney injury. *J. Am. Soc. Nephrol.* **22**, 810–820 (2011). 21493774 10.1681/ASN.2010080796

[32] Ronco, C., Bellomo, R. & Kellum, J. A. Acute kidney injury. *The Lancet* **394**, 1949–1964 (2019). 10.1016/S0140-6736(19)32563-2 31777389

[33] Ronco, C., Kellum, J. A. & Haase, M. Subclinical AKI is still AKI. *Crit. Care Lond. Engl.* **16**, 313 (2012). 10.1186/cc11240 PMC3580601 22721504

[34] Gocze, I. *et al.* Urinary biomarkers TIMP-2 and IGFBP7 early predict acute kidney injury after major surgery. *PLoS ONE* **10**, e0120863 (2015). 25798585 10.1371/journal.pone.0120863 PMC4370650

[35] Meersch, M. *et al.* Prevention of cardiac surgery-associated AKI by implementing the KDIGO guidelines in high risk patients identified by biomarkers: The PrevAKI randomized controlled trial. *Intens. Care Med* **43**, 1551–1561 (2017). 10.1007/s00134-016-4670-3 PMC5633630 28110412

[36] Jufar, A. H. *et al.* Renal functional reserve: From physiological phenomenon to clinical biomarker and beyond. *Am. J. Physiol. Regul. Integr. Comp. Physiol.* **319**, R690–R702 (2020). 33074016 10.1152/ajpregu.00237.2020

[37] Fawcett, W. J. & Thomas, M. Pre-operative fasting in adults and children: Clinical practice and guidelines. *Anaesthesia* **74**, 83–88 (2019). 30500064 10.1111/anae.14500

