# Supplementary material for: Biomarker-guided acute kidney injury risk assessment under liberal versus restrictive fluid therapy - the prospective-randomized MAYDAY-trial
Source: Sci Rep. 2024 Jul 24;14:17094. doi: 10.1038/s41598-024-68079-2 (PMC11269689; doi:10.1038/s41598-024-68079-2)

Supplementary Material 1:

To ensure that the significant study results regarding biomarker levels (t1) were not influenced by the preoperative eGFR values, we conducted a correlation analysis between these variables. The analysis showed no significant correlation between preoperative eGFR and the biomarker levels at t1 in neither the LFA group (Pearson's correlation coefficient 0.366, p=0.136), nor the FRV group (Pearson's correlation coefficient 0.084, p=0.749). This confirms that the observed biomarker differences are unlikely to be driven by the differences in preoperative eGFR.

Figure a: Scatter plots for both study groups LFA and FRV displaying t1 [TIMP-2]•[IGFBP7] biomarker levels and preoperative eGFR baseline values. *LFA* liberal fluid administration, *FRV* fluid restriction vasopressor support.), *t1* immediately postoperatively.

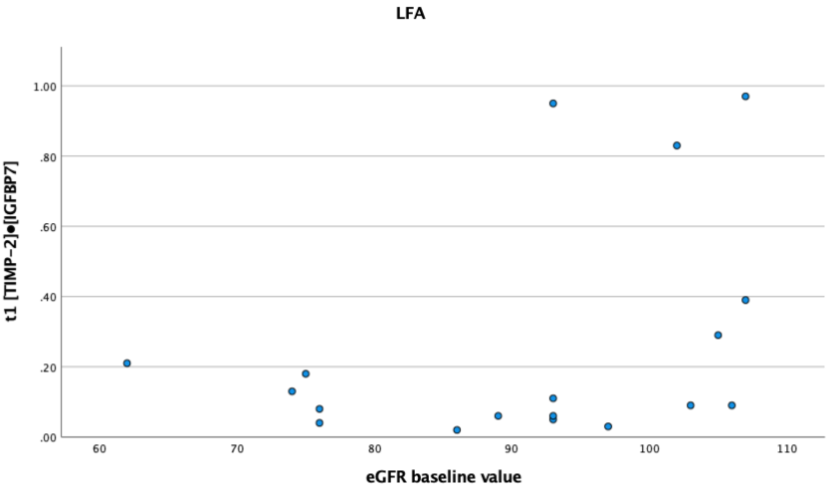

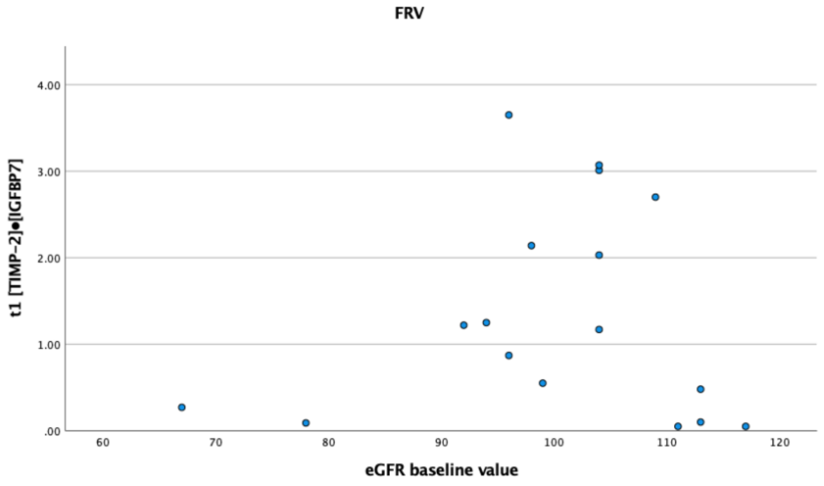

Supplement: Supplementary file 1 — Supplementary Information. [file 41598_2024_68079_MOESM1_ESM.pdf]
